# Plastic Surgery for the Oncological Patient

**DOI:** 10.3389/fsurg.2014.00042

**Published:** 2014-10-20

**Authors:** Adrien Daigeler, Kamran Harati, Nicolai Kapalschinski, Ole Goertz, Tobias Hirsch, Marcus Lehnhardt, Jonas Kolbenschlag

**Affiliations:** ^1^Department of Plastic Surgery, BG University Hospital Bergmannsheil, Ruhr-Universität Bochum, Bochum, Germany

**Keywords:** plastic surgery, reconstruction, oncology, palliative care

## Abstract

The therapy of oncological patients has seen tremendous progress in the last decades. For most entities, it has been possible to improve the survival as well as the quality of life of the affected patients. To supply optimal cancer care, a multidisciplinary approach is vital. Together with oncologists, radiotherapists and other physicians, plastic surgeons can contribute to providing such care in all stages of treatment. From biopsies to the resection of advanced tumors, the coverage of the resulting defects and even palliative care, plastic surgery techniques can help to improve survival and quality of life as well as mitigate negative effects of radiation or the problems arising from exulcerating tumors in a palliative setting. This article aims to present the mentioned possibilities by illustrating selected cases and reviewing the literature. Especially in oncological patients, restoring their quality of life with the highest patient safety possible is of utmost importance.

## Introduction

Both surgical and non-surgical treatments for malignancies have seen tremendous progress and innovations over the last decades. Due to these innovations, survival rates have improved for most cancer entities. While the search for even more potent cancer treatments is ongoing, the quality of life of the patients has come into focus. Due to the various cancer entities and inter-individual differences between patients, therapy needs to be tailored to the individual patients to create an optimal balance between effectiveness and adverse effects. To provide such an individualized therapy and the best care for oncological patients, an inter-disciplinary approach is vital. Together with oncologists, radiotherapists, physicians, and other surgical subspecialists, plastic surgeons can add valuable tools to the armamentarium of oncological care. Plastic surgery techniques can be used to cover defects following tumor resection and thereby make resections possible that otherwise would not be compatible with life, such as thoracic wall resections. Proper planning and the application of reconstructive procedures secure the adequate soft-tissue coverage and can also partly restore lost function. By augmenting the soft-tissue envelope, flap coverage can enable a timely start of radiation therapy or can be used to mitigate its adverse effects by transferring healthy tissue in case of radiation ulcers. Techniques must be as safe as possible for the patient to keep delayed wound healing to a minimum and thus not to postpone the onset of chemo- or radiation therapy. In tumors of the extremity, these techniques allow for oncological resection and functional limb salvage. By applying advanced resection techniques like intra-thoraco-scapular amputations or hemipelvectomies, even extremely extended tumors can be removed without compromising oncological safety and defects can be covered by remaining soft-tissue flaps from the amputated extremity. In situations where a complete resection of the tumor cannot be achieved by the use of these techniques, plastic surgery can help to close ulcerating wounds, facilitate patient care, and improve the quality of life of the palliative patient. This article aims to present the aforementioned possibilities drawing on a series of cases from our institution and a review of the literature.

## Resection

**Figure 1:**
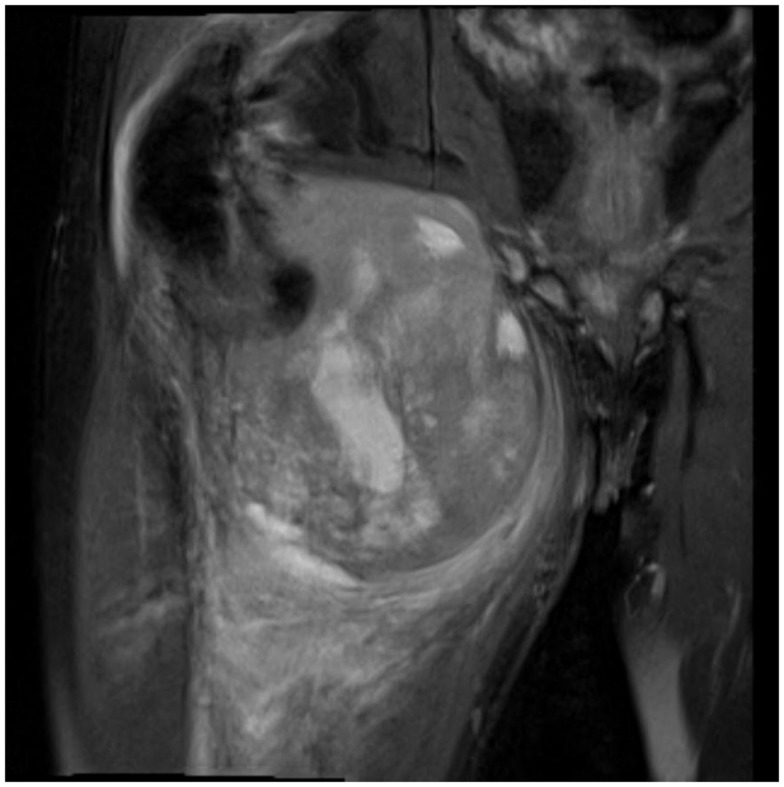
**MRI of a soft-tissue sarcoma of the thigh, reaching up to the pelvic bones**.

**Figure 2:**
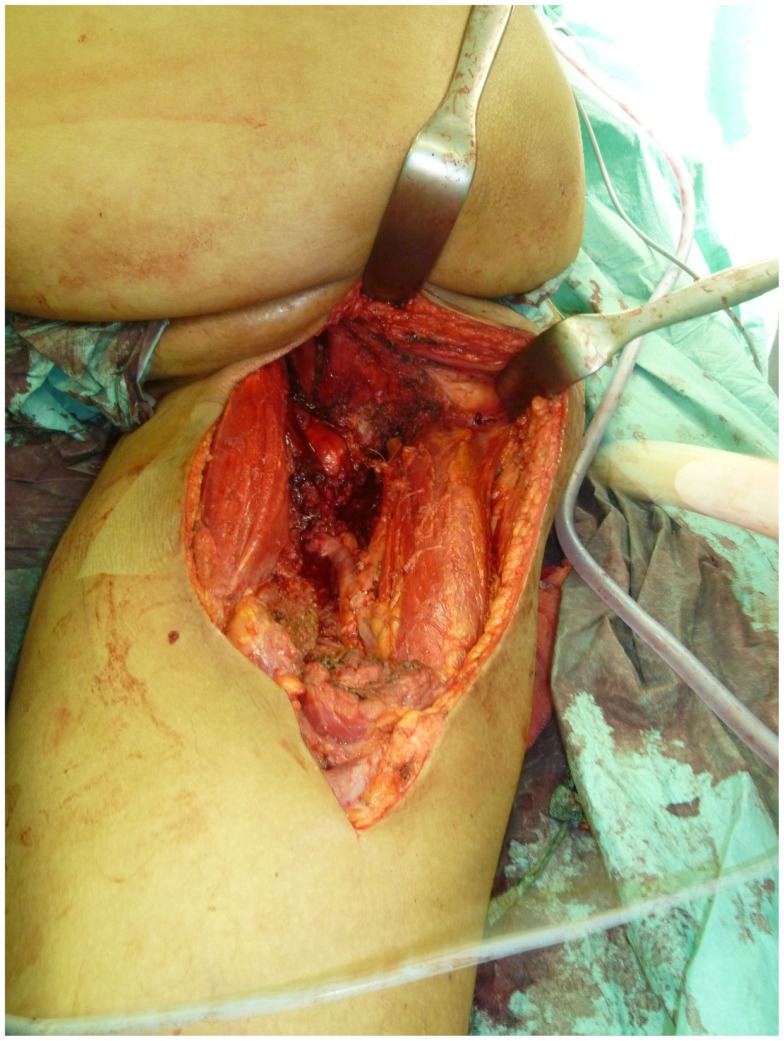
**Intraoperative situs after tumor resection with exposed gemelli muscles, tuber ischiadicum, and femoral vessels after resection of the adductor muscles**.

Not only reconstructive techniques but also the way resections of tumors are performed have changed dramatically over the last decades. In soft-tissue sarcomas, for example, we have seen a shift of the paradigms regarding the width of surgical resection, from limb amputation over resections of the whole compartment to marginal resections (1–3). Although the topic of amputation vs. limb salvage remains controversial to this date regarding the quality of life of the patients, limb salvage leads to lower lifetime medical costs and high rates of independent mobility, especially in older patients (1, 4–7). The case of a patient with an advanced soft-tissue sarcoma (not otherwise specified, NOS) of the thigh and pelvic region is depicted in Figures 1 and 2. In this case, a potentially curative resection with clear surgical margins has been achieved despite the size and location of the tumor. To achieve wider margins, a hemipelvectomy would have been required, but according to our data more radical surgery with wider margins would not improve survival but severely compromise life quality (2, 8).

**Figure 3:**
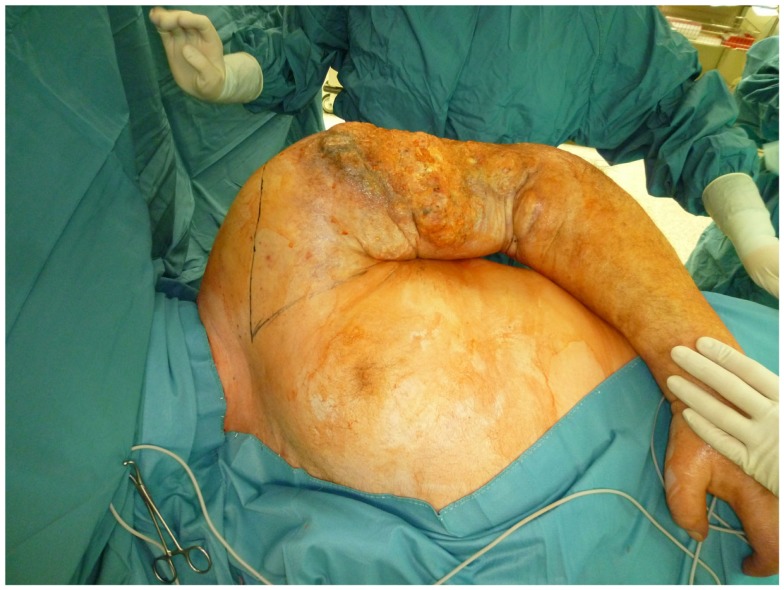
**Intraoperative aspect of the advanced carcinoma of the upper arm including markings for the planned resection**.

**Figure 4:**
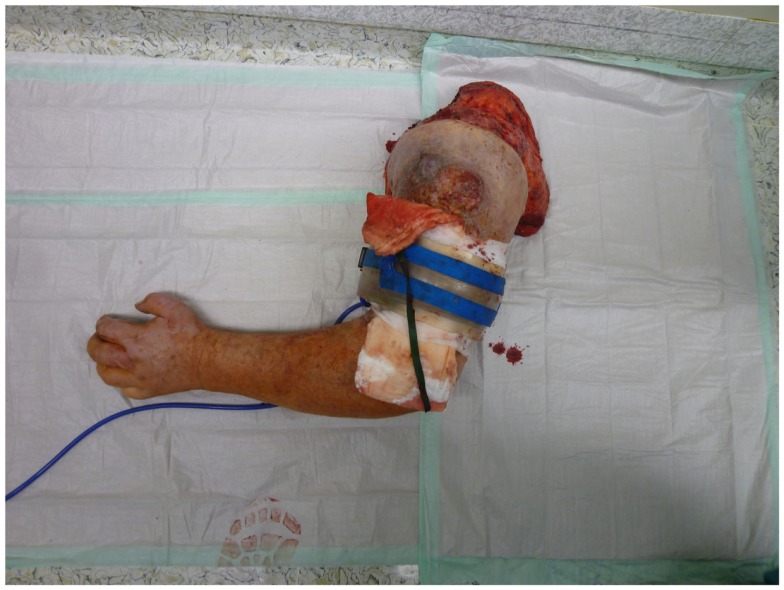
**The resected arm including the shoulder girdle at interscapulothoracic level**.

Nonetheless, in tumors beyond the reach of limb salvage, proximal major limb amputations are still to be considered an option, especially in cases with severe pain, pending vascular arrosion, bleeding, ulceration, and the loss of function of the extremity. Locally advanced tumors of the shoulder girdle can be treated by interscapulothoracic amputations (ISTA) or hemipelvectomies. Since the associated morbidities are numerous and the survival of these patients is limited, these techniques represent the last option and require a thorough patient education (8). In some of these cases, the resulting defect can be covered with free filet flaps from the amputated extremity to reduce the strain after such operations at least to some extent (9). Figures 3 and 4 show the case of a patient with advanced carcinoma of the upper arm with pain and loss of function. In order to achieve a curative resection, an ISTA had to be performed and the direct closure as well as clean margins has been achieved.

## Stump Modifications

**Figure 5:**
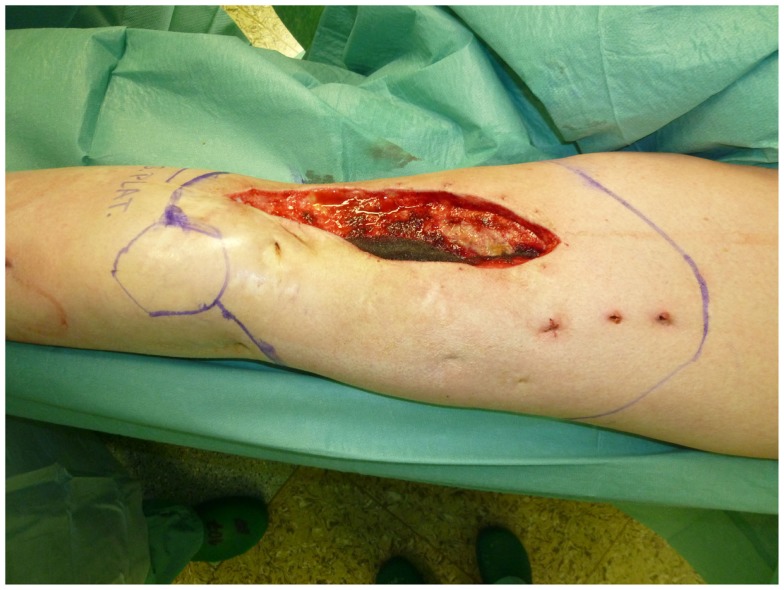
**Intraoperative aspect of an ulcerated carcinoma of the thigh**.

**Figure 6:**
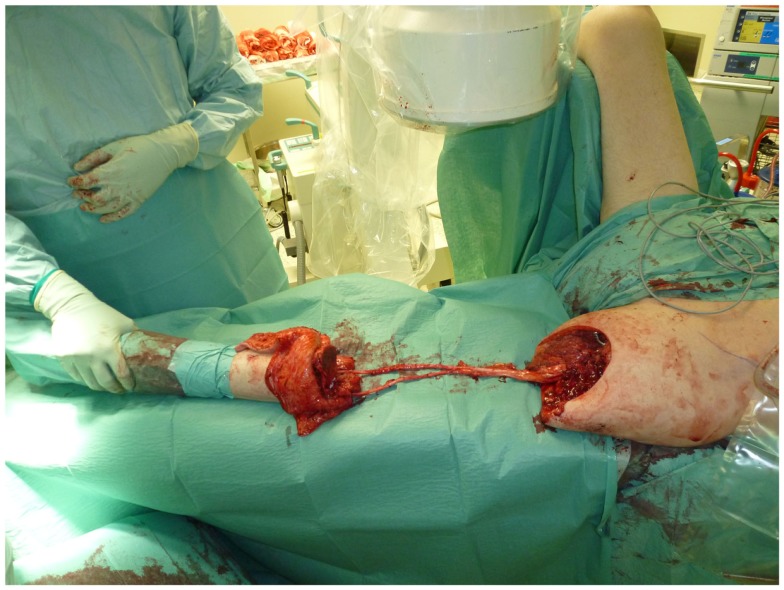
**Situs after resection of the tumor with preserved ischiadic nerve**.

**Figure 7:**
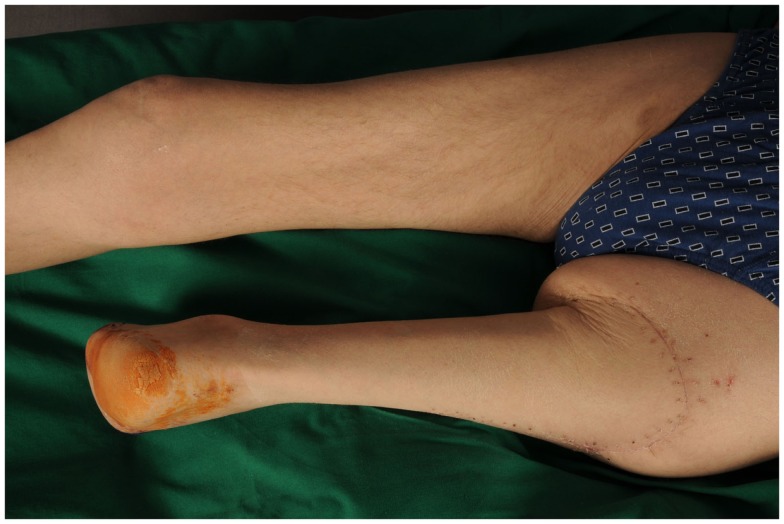
**Status four weeks post-operatively with the heel forming the new knee**.

Even despite technical progress and multimodal treatments, such extended resections as described above are still required in selected cases. In some of these cases, resection and reconstruction can be combined by means of spare part surgery. In selected malignant tumors of the leg, a Borggreve rotationplasty allows for a radical resection of the tumor while it simultaneously enables the surgeon to obtain a maximal stump length and a sensible and resilient skin cover as well as a neo-knee joint that enables the active motion of the orthesis. This makes it possible to mobilize the patient more easily by reducing wound healing disorders of the stump and the amount of energy needed for ambulation (10, 11). A case of a patient with ulcerated carcinoma affecting the femur and femoral vessels but not the ischiadic nerve is shown in Figures 5–7. In this case, a Borggreve rotationplasty has been performed after tumor resection.

Although such radical and often mutilating surgical interventions are necessary in selected cases, modern reconstructive techniques are able to close the gap between oncological safety and extremity salvage for most patients. Defects of the extremities, which would have led to amputation a few decades ago, can now be safely covered by free tissue transfers, thus salvaging the extremity. The ability to reconstruct large and full-thickness defects of the thoracic wall by reliable flaps permits radical resections, which otherwise would have been lethal. Thus, in cases where an adequate resection with clean margins and a safe reconstruction can be achieved, the preservation of form and function should be of utmost importance. Due to the steady evolution of reconstructive techniques, these aims can be achieved in most cases and with little donor-site morbidity.

Not only the operative techniques are evolving, but the settings in which the treatment of oncological patients takes place are also changing. Further specialization and inter-disciplinary teamwork has led to new sub-specialties like breast surgery, which has seen a tremendous increase in recent years (12). Due to the focused field of care and an inter-disciplinary approach, care for patients with breast malignancies can be optimized. Proper (neo-)adjuvant treatment can be tailored to the individual patient and administered in conjunction with the surgical approach. Depending on the tumor size and localization, a one-stage oncoplastic operation can be sufficient to achieve clean surgical margins and a good cosmetic result without compromising oncological safety. In more advanced malignancies of the breast leading to its amputation, modern reconstructive techniques like perforator-based flaps from the abdomen or the buttock allow for an esthetic reconstruction with autologous tissue and little donor-site morbidity. Therefore, to achieve optimal results, the tumor resection needs to be planned with the reconstruction in mind. This is also true for incisional biopsies in larger tumors, where both the surgical incision and a potential drain needs to be placed under consideration of a following resection.

Due to this wide variety of factors that need to be considered to deliver optimal cancer care, specialized centers with an inter-disciplinary approach have continued to emerge. Such specialized high-volume centers can improve the overall outcome of cancer operations due to the specialists’ experience, the clinical routine and the close collaboration of all disciplines involved (13, 14). These do not only include surgical and oncological disciplines but also a specialized department of pathology with adequate experience in the center’s special field of cancer as well as dedicated physical and occupational therapists to enable a fast recovery of the patient.

## Reconstruction

Due to the plethora of reconstructive possibilities available today, a comprehensive description is impossible to deliver within the limited scope available. Therefore, we aim to present a general pattern of indications and reconstructive possibilities for common problems in oncological patients.

Although technical improvements have led to an increased armamentarium of flaps, all available techniques should be carefully considered when planning a (microsurgical) reconstruction. Among the factors that need to be considered are the location and size of the defect, potential receiving vessels and the required pedicle length, relevant comorbidities and past operations, and the individual distribution of soft-tissue to avoid an excessive bulkiness of the transplanted tissue, especially on the extremities.

Amid the wealth of reconstructive possibilities available for a specific problem, the question of donor-site morbidity and hence the patients’ quality of life also needs to be considered.

To choose an adequate reconstructive procedure, the basic principle of the reconstructive ladder can be applied. The reconstructive ladder illustrates the increasing technical demand from simple sutures to skin grafting, local, and pedicled flaps up to microvascular tissue transfer. Based on this principle, the safest and least challenging method that still offers sufficient coverage of the defect should be chosen. Based on the factors mentioned above and with the reconstructive ladder in mind, one can choose the most suitable reconstructive procedure for a particular case. Although the reconstructive ladder provides good guidance for most cases, it can be necessary in selected cases to skip some of its steps. For example, in a patient with radiation damage, coverage via directly adjacent flaps is often difficult to achieve due to the resulting fibrosis of the soft-tissues. In such cases, free flaps should be considered even for smaller defects, since the unaffected tissue can be safely applied in radiated tissue or before radiation therapy and can even mitigate the adverse effects of radiotherapy such as unstable skin cover or scarring.

## Pedicled Flaps

Pedicled flaps are based on various compositions of tissue with a dedicated blood supply. By maintaining this blood supply and transposing the dissected flap, a nearby defect can be closed. Due to the inherent blood supply and the amount of tissue that can be transferred, such flaps can reconstruct defects with exposed functional structures like tendons or bone, which cannot be covered by skin grafts.

Pedicled flaps are readily available at the ventral trunk and are sufficient for most reconstructive challenges. On the extremities, flap coverage is often required due to the relatively little soft-tissue coverage and bony prominences. While there are several working horse flaps for the local reconstruction of soft-tissue defects of the lower extremity, large defects of these areas frequently require free tissue transfer.

## Latissimus Dorsi Flap

A pedicled latissimus dorsi flap based on the thoraco-dorsal artery can sufficiently cover most defects on the thoracic wall. It can be harvested with relative ease due to the large caliber vessels and its anatomical reliability. Due to its large radius of rotation, including the ventral thoracic wall, the sternal region and the upper arm, it is a versatile flap that can cover large defects. Except for a relatively high rate of seroma formation at the donor site, which can be treated conservatively in most cases, the donor-site morbidity is low (15). It can also be harvested as a free flap and is especially valuable for covering large defects.

## Rectus Abdominis Flap

**Figure 8:**
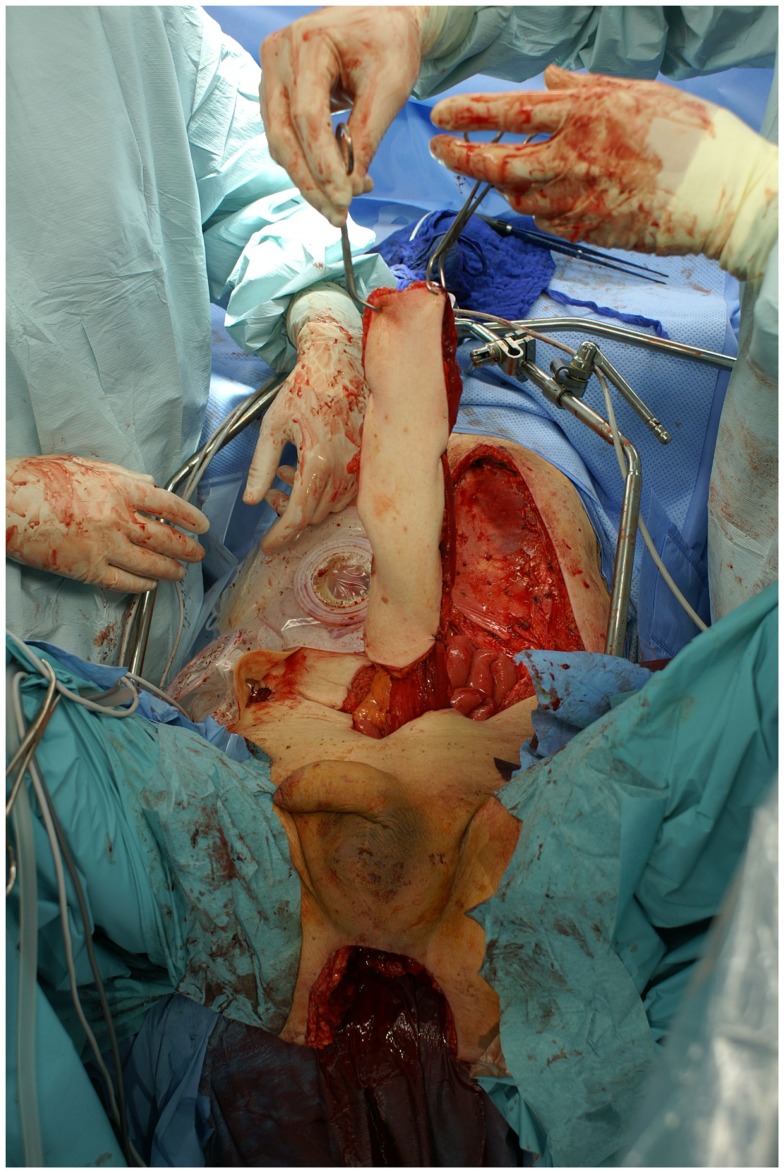
**Intraoperative aspect of the pedicled VRAM flap prepared to cover a perineal defect**.

**Figure 9:**
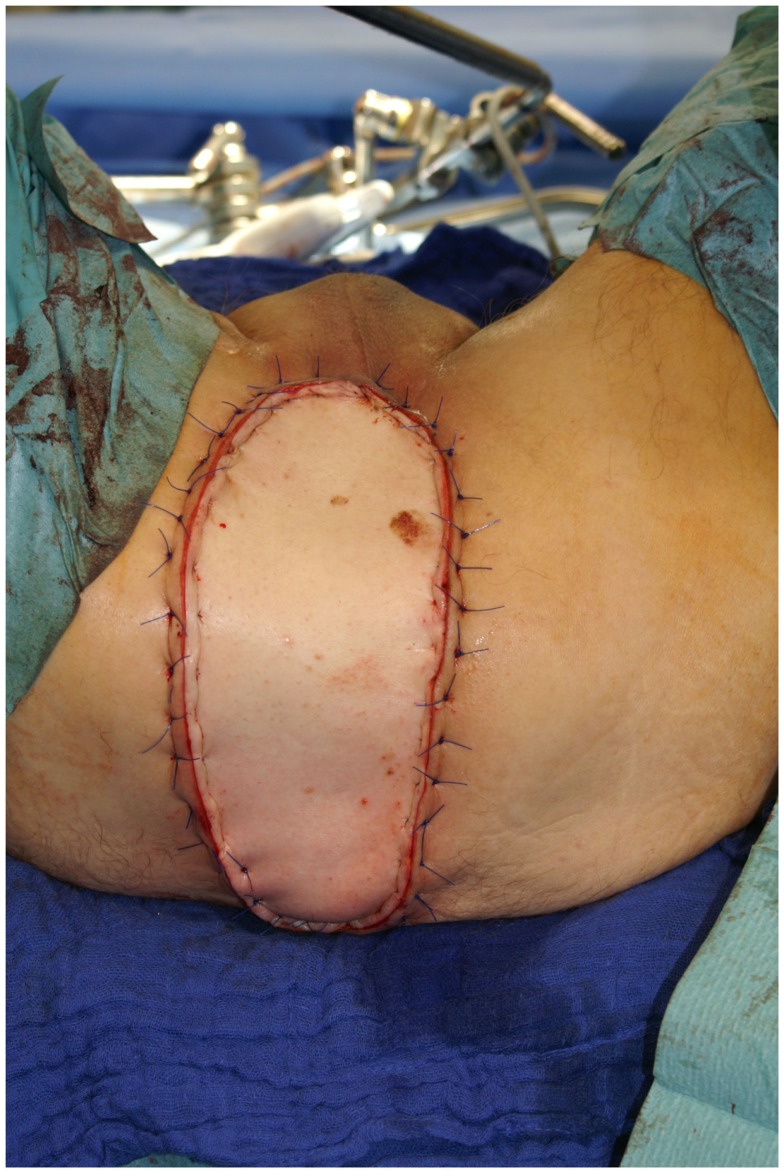
**Situation after transabdominal transposition of the flap**.

Another versatile pedicled flap that can be harvested with relative ease is the rectus abdominis muscle flap. Utilizing either a vertical (vertical rectus abdominis muscle flap; VRAM) or a transverse (TRAM) skin island, it can be harvested with a cranial or caudal pedicle. This greatly increases the flap’s range of transposition and therefore its versatility. Based on a cranial pedicle, a VRAM can easily cover the whole sternal region and the upper thorax. Utilizing a caudal pedicle, this flap allows for the coverage of the inguinal region and can also help to reduce the lymphatic congestion that often goes along with an extended tumor resection in the inguinal region by drainage to the contralateral side (16). It can also be tunneled through the pelvis and be used to close perineal defects after the resection of rectal cancer. The VRAM and TRAM can be combined as an “anchor flap” to allow for the coverage of very large defects, e.g., after the radical resection of advanced malignancies of the anterior chest wall. Figures 8 and 9 depict the case of a male patient undergoing a VRAM procedure for the perineal reconstruction following the resection of advanced rectal cancer.

## Gastrocnemius Muscle Flap

**Figure 10:**
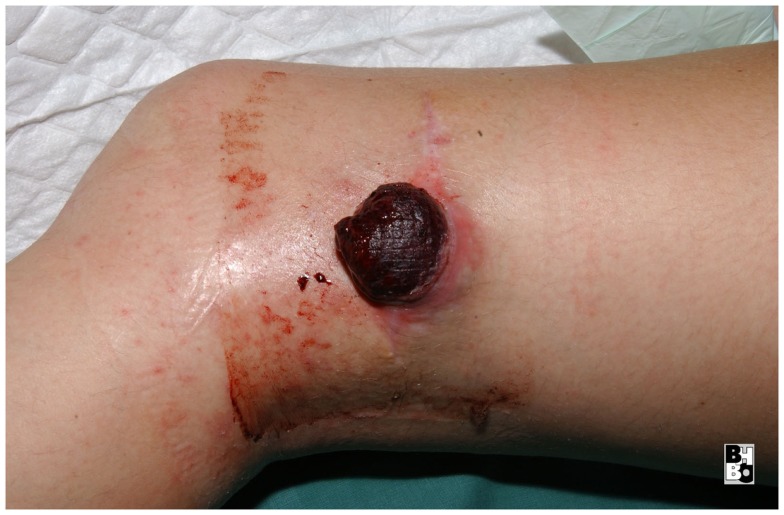
**Preoperative aspect of an ulcerating sarcoma of the lateral knee**.

**Figure 11:**
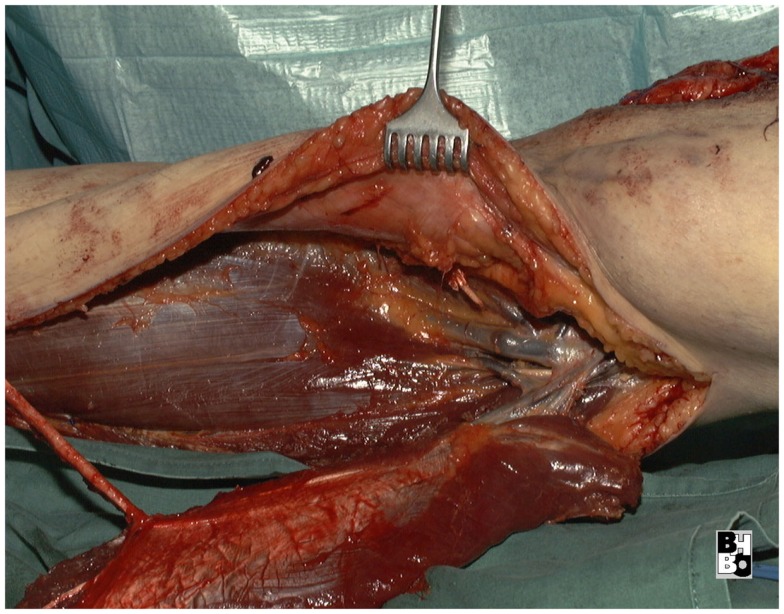
**Intraoperative aspect after resection of the tumor and elevation of the pedicled lateral gastrocnemius muscle flap**. Notice the dissected vascular pedicle of the flap.

**Figure 12:**
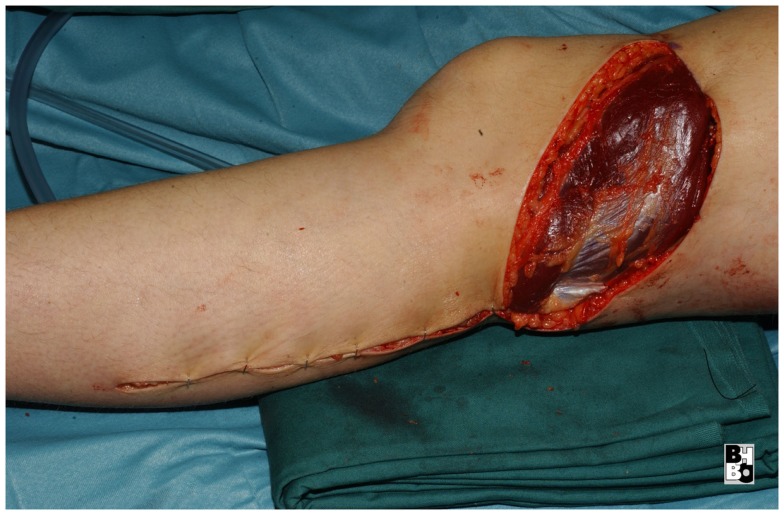
**After transposing the flap into the defect and primary closure of the donor site**. The flap still requires a split skin graft.

The gastrocnemius muscle flap is a safe and reliable flap that can easily cover the knee and the proximal tibia. It supplies well perfused and thick muscular tissue that creates a durable and resilient cover but requires skin grafting to cover the muscle. The donor-site morbidity is moderate and well tolerated by the majority of patients (17, 18). Figures 10–12 show a case of an exulcerating sarcoma of the lateral knee. After the resection of the tumor, the resulting defect is covered with a pedicled lateral gastrocnemius flap and a skin graft.

## Pedicled Propeller Flaps

Another recent development is the so-called propeller flaps. With conventional pedicled flaps, one is limited to certain given donor sites where flaps based on an axial blood supply can be raised, e.g., the latissimus dorsi flap or the sural artery flap. Depending on their blood supply, such flaps can be harvested in either an antegrade or retrograde fashion, which can extend their range of transposition. While there is a variety of pedicled flaps available in certain anatomic regions (e.g., the ventral thoracic wall), such opportunities are rare in other regions (e.g., the dorsal trunk). Based on the perforator principle discussed above, one can identify a single perforating vessel via hand-held doppler and can design a flap based on this vessel. To enable a wider range of rotation, the vessel can be placed in an eccentric position and the raised flap can be rotated up to 180° to close adjacent defects, which explains the term “propeller flap.” Such flaps are seeing an increased clinical application, especially in regions where “conventional” pedicled flaps are sparely available (19). Due to their comparable skin texture and appearance as well as less bulk, such flaps can provide better esthetic results. Achieving such good esthetic results and the best quality of life possible is a high objective. However, as with the free flaps discussed later, this may not lead to neglecting the safety of the technique and therefore the patients’ safety.

## Free Flaps

Free flaps are based on a dedicated blood supply by well-defined vessels. Using these vessels, the flap can be dissected free from the surrounding tissue and transplanted to a remote defect, where the flap’s vessels can be anastomosed to a local recipient vessel by means of a surgical microscope. Such flaps can incorporate various tissues from skin and subcutaneous tissue to muscle and tendons and can be harvested in various sizes. Since the choice of the right flap is not limited to the region of the defect, a variety of flaps is available for microvascular reconstruction. Therefore, the optimal flap for a certain defect can be chosen with great liberty, taking different factors into account like the size of the defect, its location, and available recipient vessels as well as the resulting donor-site morbidity from the flap harvest.

## TRAM/MS-TRAM/DIEP

**Figure 13:**
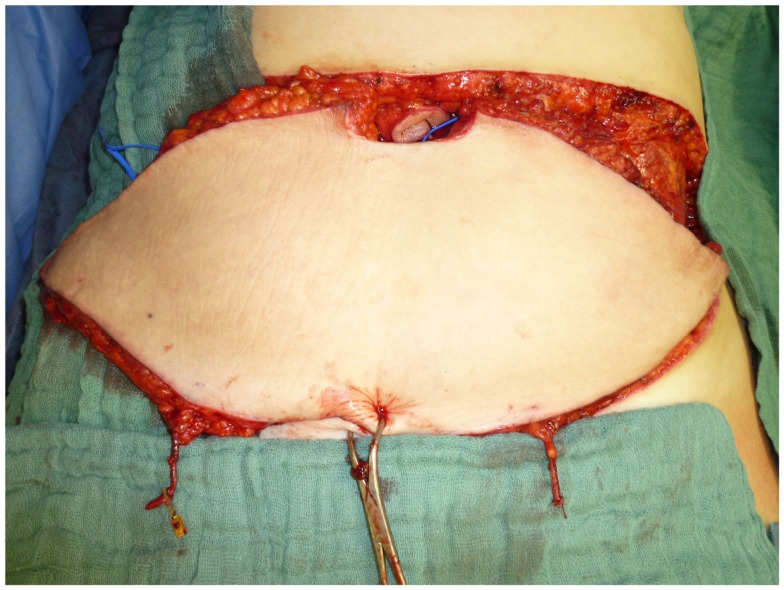
**Intraoperative aspect after harvesting the flap**. Notice the two dissected perforating vessels that provide the flap’s blood supply.

**Figure 14:**
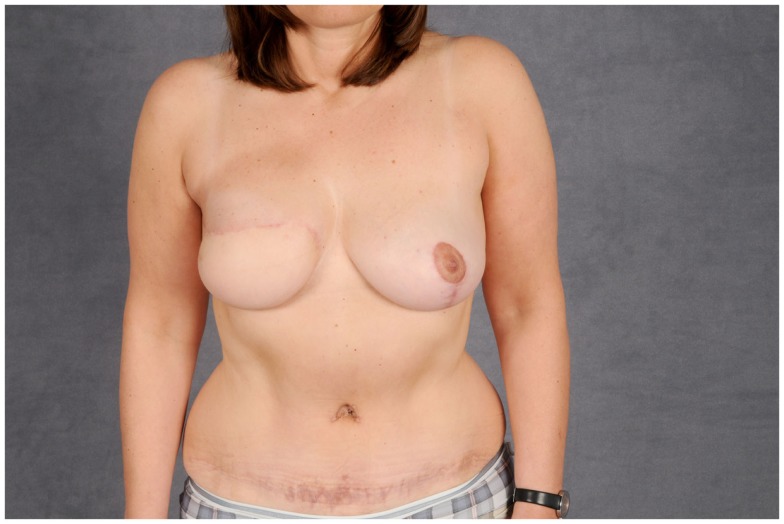
**Post-operative aspect of the same patient**. The abdominal defect is closed by an abdominoplasty.

The TRAM flap described above can also be harvested as a free flap and was once the mainstay for autologous breast reconstruction due to the bulk it can deliver and the relatively easy technique. The donor-site morbidity after removal included abdominal wall weakness and symptomatic herniation. To mitigate this significant downside, the amount of muscle removed was reduced to a cuff around the vascular pedicle, resulting in the muscle-sparing rectus abdominis flap (MS-TRAM). In a further attempt to reduce donor-site morbidity, the perforating vessel supplying the skin over the lower abdomen was dissected through the muscle to its origin at the deep inferior epigastric artery. This resulted in the deep inferior epigastric artery perforator flap (DIEP), which has become one of the most popular choices for autologous breast reconstruction today with only moderate donor-site morbidity. Figures 13 and 14 depict the case of a patient undergoing DIEP flap reconstruction following the ablation of the right breast due to carcinoma.

## Lymphatic Surgery

A common problem after tumor resection and lymph node dissection both in the axilla and the groin area is a persistent lymph edema of the affected extremity. While conservative treatment with manual lymph therapy and pressure garments can improve this condition, it often remains a serious concern for the patients. In recent years, various operative treatments for lymph edema have emerged. The most established procedures are based on creating lymphovenous shunts or on transplanting lymph nodes from remote regions.

In both approaches, microsurgical techniques are employed to improve lympathic drainage via the venous system or by the reconstruction and augmentation of the lympathic system.

While both approaches seem to yield good results, the transplantation of lymph nodes has shown better overall results, at least according to the few studies available today (20–22).

In patients undergoing breast reconstruction with DIEP flaps, these reconstructive procedures can be combined by including inguinal lymph nodes into the DIEP flap for a reconstruction of the axillary drainage.

## Anterior Lateral Thigh

This principle of using the perforating vessels that supply a dedicated area of skin and subcutaneous tissue (perforasome) as the flap’s vascular pedicle can also be applied to various other flaps (23). The anterior lateral thigh (ALT) flap is such a flap and has become one of the work-horse perforator flaps for microsurgical reconstruction due to its versatility, the relatively little bulk, and the fact that it can be raised in a prone position. It can be harvested as a fascio- or adipocutaneous flap and be used for a variety of reconstructive purposes. Although it has no obvious functional donor-site morbidity, a recent study has shown that the majority of patients who received both flaps prefer the donor-site of the parascapular flap over the ALT (24).

Also, the variable patterns of blood supply of perforator flaps need to be taken into consideration. While “classic” free flaps, like the parascapular flap, have a reliable, longitudinal blood supply with little anatomical variation and can therefore be harvested with relative ease, perforator flaps are more difficult to raise and require advanced technical skills.

## Functional Reconstruction

In cases where neuro-vascular structures or large portions of muscle tissue have to be resected, the resulting functional deficits can be attenuated by reconstructive procedures. Neural reconstructions by means of autologous transplantation or conduits often yield only abstemious results, especially in cases where adjuvant radiotherapy is applied.

Therefore, motoric reconstructions utilizing the transfer of tendons of still functioning muscles to replace the lost function are a good choice for most patients. One of the most common conditions warranting such a functional reconstruction is the proximal radial nerve palsy, due to trauma or tumor resection. Various transfers of tendons have been described for this condition, one of the most common types of reconstruction utilizes the pronator teres muscle for wrist extension, the palmaris longus muscle for thumb extension and abduction and the flexor carpi ulnaris muscle to regain finger extension. These transfers yield good functional results with relatively little donor-site morbidity (25, 26). Although the muscles that can be used vary greatly between patients and their individual patterns of functional loss, the principle of replacing lost muscle function by the transfer of still working muscles remains valid and can be applied for various conditions.

On the lower extremity, one of the most common conditions that can be treated by functional tendon transfer is the drop foot deformity resulting from injury or the resection of the fibular nerve. By transferring the posterior tibial tendon and fixing it to the anterior tibial and the long peroneal tendon as a stirrup-procedure, active dorsiflexion of the foot can be restored. This enables most patients to regain normal gait without the help of orthotic devices.

A recent study has shown that the active range of motion of the foot and the quality of life can be improved by this reliable procedure without altering the pressure distribution of the foot or a significant donor-site morbidity (27).

In patients with loss of the knee’s extensor apparatus, either due to the resection of the quadriceps muscle or the femoral nerve, the hamstring muscle tendons can be transferred to the patella or patellar tendon to regain at least a moderate amount of extension and enable a normal gait.

When the hamstring muscles are unavailable, a free muscle transfer can also yield good results, although the rehabilitation period is significantly longer than in patients with direct tendon transfers (28–31).

Functional muscle transfers also present a valid reconstructive possibility for the upper extremity. In patients undergoing a resection of the ventral upper arm compartment, the pedicled latissimus dorsi muscle can be transferred and reinserted at the lower arm to replace the biceps brachii muscle and allow for active flexion of the elbow joint. A recent study has revealed a low strength of elbow flexion but a good range of motion in these patients (32), enabling them to participate in daily activities with much less constraint.

**Figure 15:**
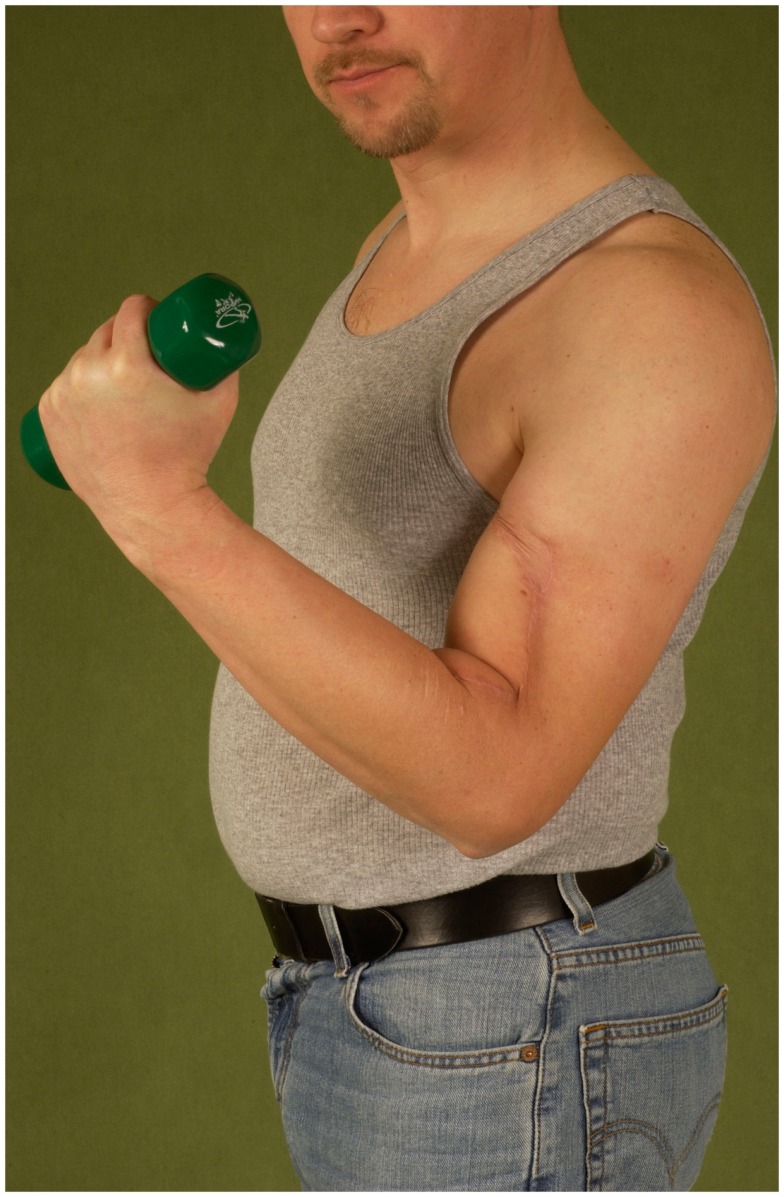
**Restored active flexion of the elbow by reinsertion of the transferred latissimus muscle to remnants of the biceps tendons**.

Figure 15 shows the case of a patient undergoing the resection of the ventral compartment of the upper arm due to an advanced soft-tissue sarcoma followed by functional reconstruction via a pedicled latissimus dorsi flap.

In cases where the option of a pedicled muscle transfer is not available, the gracilis muscle can be transferred to the upper arm and coapted to a donor nerve to restore elbow function (33).

## Palliation

Due to advances in adjuvant and neoadjuvant treatment modalities, survival rates have improved over the last decades, even for patients with advanced tumor states.

For such patients, improving the quality of life is paramount when considering their limited life expectancies. Local cancer progress can result in exulcerated, bleeding, and painful wounds that often require extensive dressings and complicated patient care. Foul odor and secretion lead to social isolation and a further decrease of the patients’ quality of life.

While a conservative treatment with wound irrigation and dressing changes is possible, it is time consuming, painful, and can only mitigate the impact of the wounds on social interaction.

In such cases, palliative tumor resections and coverage of the resulting defects can help these patients to regain their independence and enable them to socially interact again (34). In a palliative setting, the use of reliable and safe techniques is of utmost importance to reduce the duration of hospital stay for those patients with regard to their limited life expectancy. This has to be considered from the very beginning of the treatment plan.

In these settings, a compromise between the radicalness of the resection and the resulting strain for the patient needs to be found. In patients with advanced tumors with infiltration of the thoracic wall for example, whole-thickness resections are often not warranted, since they dramatically increase the invasiveness, the reconstructive effort, and the recovery time for the patient. Especially, in a palliative setting, a close, honest, and comprehensive counseling of the patient is vital. Also, post-operative care and rehabilitation needs to address special issues of these patients, such as a focus on a timely return to activities of daily life and psycho-oncological support. Based on these principles, surgery should not be shunned in the face of limited life expectancy and advanced tumors but used wisely to help patients regain their quality of life and social independence.

## Conclusion

Plastic surgery can aid in the inter-disciplinary concept of care for oncological patients by verifying the diagnosis via biopsies, the planning and execution of a tumor resection, and the coverage of the resulting defect and functional reconstructions. Even in advanced tumor stages, safe and reliable techniques allow for the removal of exulcerating tumors and timely defect coverage and enable patients to regain life quality. Safe and reliable procedures with a clear focus on the patients’ safety and their quality of life are essential to provide optimal care for oncological patients. While oncological safety remains of utmost importance, a multimodal approach and advanced plastic surgery techniques can improve survival and life quality as well as mitigate resulting functional deficits.

Due to the growing complexity of oncological care and the variety of disciplines involved, this can be best achieved in an inter-disciplinary setting of close collaborations (tumor boards, joint rounds, etc.) and is therefore well suited in specialized high-volume centers.

## Conflict of Interest Statement

The authors declare that the research was conducted in the absence of any commercial or financial relationships that could be construed as a potential conflict of interest.

## References

[1] Barr RD Wunder JS Bone and soft tissue sarcomas are often curable – but at what cost?: a call to arms (and legs). Cancer (2009) 115(18):4046–54 10.1002/cncr.24458 19670445

[2] Goertz O Langer S Uthoff D Ring A Stricker I Tannapfel A Diagnosis, treatment and survival of 65 patients with malignant peripheral nerve sheath tumors. Anticancer Res (2014) 34(2):777–83. 24511012

[3] Lehnhardt M Hirche C Daigeler A Goertz O Ring A Hirsch T [Soft tissue sarcoma of the upper extremities. Analysis of factors relevant for prognosis in 160 patients]. Chirurg (2012) 83(2):143–52. 10.1007/s00104-011-2124-6 21695557

[4] Chung KC Saddawi-Konefka D Haase SC Kaul G. A cost-utility analysis of amputation versus salvage for Gustilo type IIIB and IIIC open tibial fractures. Plast Reconstr Surg (2009) 124(6):1965–73. 10.1097/PRS.0b013e3181bcf156 19952652 PMC2788746

[5] Kolbenschlag J Hellmich S Germann G Megerle K. Free tissue transfer in patients with severe peripheral arterial disease: functional outcome in reconstruction of chronic lower extremity defects. J Reconstr Microsurg (2013) 29(9):607–14. 10.1055/s-0033-1354739 24019177

[6] Steinau HU Daigeler A Langer S Steinstrasser L Hauser J Goertz O Limb salvage in malignant tumors. Semin Plast Surg (2010) 24(1):18–33 10.1055/s-0030-1253240 21286302 PMC2887000

[7] Zahlten-Hinguranage A Bernd L Sabo D. [Amputation or limb salvage? Assessing quality of life after tumor operations of the lower extremity]. Orthopäde (2003) 32(11):1020–7. 10.1007/s00132-003-0548-5 14615852

[8] Daigeler A Lehnhardt M Khadra A Hauser J Steinstraesser L Langer S Proximal major limb amputations – a retrospective analysis of 45 oncological cases. World J Surg Oncol (2009) 7:15. 10.1186/1477-7819-7-15 19203359 PMC2647924

[9] Kuntscher MV Erdmann D Homann HH Steinau HU Levin SL Germann G. The concept of fillet flaps: classification, indications, and analysis of their clinical value. Plast Reconstr Surg (2001) 108(4):885–96. 10.1097/00006534-200109150-00011 11547143

[10] Veenstra KM Sprangers MA van der Eyken JW Taminiau AH. Quality of life in survivors with a Van Ness-Borggreve rotationplasty after bone tumour resection. J Surg Oncol (2000) 73(4):192–7. 10.1002/(SICI)1096-9098(200004)73:4192::AID-JSO23.0.CO;2-H 10797331

[11] Homann HH Lehnhardt M Langer S Steinau HU. [Stump retention and extension on the lower extremity]. Chirurg (2007) 78(4):308–15. 10.1007/s00104-007-1313-9 17356829

[12] Quinn McGlothin TD. Breast surgery as a specialized practice. Am J Surg (2005) 190(2):264–8. 10.1016/j.amjsurg.2005.05.024 16023443

[13] Lehnhardt M Daigeler A Homann HH Hauser J Langer S Steinstrasser L [Importance of specialized centers in diagnosis and treatment of extremity-soft tissue sarcomas. Review of 603 cases]. Chirurg (2009) 80(4):341–7. 10.1007/s00104-008-1562-2 18523742

[14] Rose M Manjer J Ringberg A Svensson H. Surgical strategy, methods of reconstruction, surgical margins and postoperative complications in oncoplastic breast surgery. Eur J Plast Surg (2014) 37:205–14. 10.1007/s00238-013-0922-4 24659858 PMC3950564

[15] Fischer S Klinkenberg M Behr B Hirsch T Kremer T Hernekamp F Comparison of donor-site morbidity and satisfaction between anterolateral thigh and parascapular free flaps in the same patient. J Reconstr Microsurg (2013) 29(8):537–44. 10.1055/s-0033-1351394 23982858

[16] Daigeler A Simidjiiska-Belyaeva M Drucke D Goertz O Hirsch T Soimaru C The versatility of the pedicled vertical rectus abdominis myocutaneous flap in oncologic patients. Langenbecks Arch Surg (2011) 396(8):1271–9. 10.1007/s00423-011-0823-6 21779830

[17] Daigeler A Drucke D Tatar K Homann HH Goertz O Tilkorn D The pedicled gastrocnemius muscle flap: a review of 218 cases. Plast Reconstr Surg (2009) 123(1):250–7. 10.1097/PRS.0b013e3181904e2e 19116559

[18] Suda AJ Cieslik A Grützner PA Münzberg M Heppert V. Flaps for closure of soft tissue defects in infected revision knee arthroplasty. Int Orthop (2014) 38(7):1387–92. 10.1007/s00264-014-2316-z 24663397 PMC4071491

[19] Behr B Hirsch T Goertz O Ring A Lehnhardt M Daigeler A. [Therapeutic options for reconstruction of the dorsal trunk wall]. Handchir Mikrochir Plast Chir (2014) 46(2):90–6. 10.1055/s-0034-1370994 24777458

[20] Basta MN Gao LL Wu LC Operative treatment of peripheral lymphedema: a systematic meta-analysis of the efficacy and safety of lymphovenous microsurgery and tissue transplantation. Plast Reconstr Surg (2014) 133(4):905–13 10.1097/PRS.0000000000000010 24352208

[21] Boccardo F Fulcheri E Villa G Molinari L Campisi C Dessalvi S Lymphatic microsurgery to treat lymphedema: techniques and indications for better results. Ann Plast Surg (2013) 71(2):191–5. 10.1097/SAP.0b013e31824f20d4 23542829

[22] Cheng MH Chen SC Henry SL Tan BK Lin MC Huang JJ Vascularized groin lymph node flap transfer for postmastectomy upper limb lymphedema: flap anatomy, recipient sites, and outcomes. Plast Reconstr Surg (2013) 131(6):1286–98 10.1097/PRS.0b013e31828bd3b3 23714790

[23] Saint-Cyr M Wong C Schaverien M Mojallal A Rohrich RJ. The perforasome theory: vascular anatomy and clinical implications. Plast Reconstr Surg (2009) 124(5):1529–44. 10.1097/PRS.0b013e3181b98a6c 20009839

[24] Klinkenberg M Fischer S Kremer T Hernekamp F Lehnhardt M Daigeler A. Comparison of anterolateral thigh, lateral arm, and parascapular free flaps with regard to donor-site morbidity and aesthetic and functional outcomes. Plast Reconstr Surg (2013) 131(2):293–302. 10.1097/PRS.0b013e31827786bc 23357991

[25] Seiler JG III Desai MJ Payne SH. Tendon transfers for radial, median, and ulnar nerve palsy. J Am Acad Orthop Surg (2013) 21(11):675–84. 10.5435/JAAOS-21-11-675 24187037

[26] Moussavi AA Saied A Karbalaeikhani A. Outcome of tendon transfer for radial nerve paralysis: comparison of three methods. Indian J Orthop (2011) 45(6):558–62. 10.4103/0019-5413.87133 22144751 PMC3227362

[27] Steinau HU Tofaute A Huellmann K Goertz O Lehnhardt M Kammler J Tendon transfers for drop foot correction: long-term results including quality of life assessment, and dynamometric and pedobarographic measurements. Arch Orthop Trauma Surg (2011) 131(7):903–10. 10.1007/s00402-010-1231-z 21246379

[28] Vogt PM Knobloch K Local tendon transfer for knee extensor mechanism reconstruction. Microsurgery (2009) 29(7):584–5 10.1002/micr.20679 19530084

[29] Fansa H Meric C [Reconstruction of quadriceps femoris muscle function with muscle transfer]. Handchir Mikrochir Plast Chir (2010) 42(4):233–8 10.1055/s-0030-1248311 20235008

[30] Wechselberger G Ninkovic M Pulzl P Schoeller T. Free functional rectus femoris muscle transfer for restoration of knee extension and defect coverage after trauma. J Plast Reconstr Aesthet Surg (2006) 59(9):994–8. 10.1016/j.bjps.2005.12.030 16920595

[31] Hierner R Reynders-Frederix P Bellemans J Stuyck J Peeters W. Free myocutaneous latissimus dorsi flap transfer in total knee arthroplasty. J Plast Reconstr Aesthet Surg (2009) 62(12):1692–700. 10.1016/j.bjps.2008.07.038 19071073

[32] Cambon-Binder A Belkheyar Z Durand S Rantissi M Oberlin C. Elbow flexion restoration using pedicled latissimus dorsi transfer in seven cases. Chir Main (2012) 31(6):324–30. 10.1016/j.main.2012.10.169 23177904

[33] Barrie KA Steinmann SP Shin AY Spinner RJ Bishop AT. Gracilis free muscle transfer for restoration of function after complete brachial plexus avulsion. Neurosurg Focus (2004) 16(5):E8. 10.3171/foc.2004.16.5.9 15174828

[34] Kippenhan T Hirche C Lehnhardt M Daigeler A. [Palliative plastic surgery in multidisciplinary therapeutic concepts.]. Zentralbl Chir (2013). 10.1055/s-0032-1328184 23696204

